# Optimization of Loop-Mediated Isothermal Amplification (LAMP) for the Rapid Detection of Nosocomial Pathogens on Environmental Surfaces

**DOI:** 10.3390/ijms26135933

**Published:** 2025-06-20

**Authors:** Federica Marino, Caterina Bonincontro, Laura Caligaris, Letizia Bellucci, Carlo Derelitto, Luna Girolamini, Sandra Cristino

**Affiliations:** 1 Department of Biological, Geological, and Environmental Sciences, University of Bologna, 40126 Bologna, Italy; federica.marino20@unibo.it (F.M.); caterina.bonincontr2@unibo.it (C.B.); laura.caligaris2@unibo.it (L.C.); carlo.derelitto@unibo.it (C.D.); luna.girolamini2@unibo.it (L.G.); 2 Class S.r.l., 40054 Budrio, Italy; letizia.bellucci@class.srl

**Keywords:** loop-mediated isothermal amplification (LAMP), nosocomial pathogens, surface microbiological monitoring, rapid pathogen detection, real-time environmental monitoring

## Abstract

Contamination of environmental surfaces by nosocomial pathogens like *Pseudomonas aeruginosa* (*P. aeruginosa*), *Staphylococcus aureus* (*S. aureus*), and *Enterococcus* spp. poses significant health risks worldwide. However, gold-standard detection methods are too time-consuming and labor-intensive. This study aimed to optimize loop-mediated isothermal amplification (LAMP) as a rapid, innovative, and cost-effective approach, comparing its effectiveness with the gold-standard cultural method. Sterile surfaces (24 cm^2^) were contaminated in duplicate with different concentrations of *P. aeruginosa*, *S. aureus*, and *Enterococcus faecalis (E. faecalis)* reference stains. For each pair of contaminated surfaces, one was analyzed using the agar contact plate method (UNI EN 17141:2021), while the other was analyzed using LAMP, following three different pre-incubation times (three, six, and nine hours). The sensitivity and accuracy of LAMP for *P. aeruginosa* improved with longer incubation times, reaching a value of 1.00 at nine hours, while the specificity and positive predictive value (PPV) remained at 1.00 regardless of the incubation time. For *S. aureus*, LAMP achieved a sensitivity, specificity, accuracy, PPV, and negative predictive value (NPV) of 1.00 across all incubation times. Finally, for *E. faecalis*, sensitivity increased from 0.57 at three hours to 1.00 at six and nine hours, with a high specificity, accuracy, PPV, and NPV from six hours onwards. These findings showed that LAMP can be used as a rapid and reliable alternative to gold-standard methods for detecting pathogens on surfaces. The high sensitivity and specificity achieved, especially at six and nine hours of pre-incubation, suggested its use for real-time monitoring in healthcare settings. Further research in real-world environments is needed to confirm these findings.

## 1. Introduction

The contamination of environmental surfaces by pathogenic microorganisms poses a serious public health issue, particularly in hospital settings and healthcare facilities, where the risk of infection is markedly heightened [1,2]. These surfaces could indeed play a crucial role in the transmission of nosocomial pathogens, especially for patients with compromised immune systems [3,4]. Among the most common pathogens found on hospital surfaces, *Pseudomonas aeruginosa* (*P. aeruginosa*), *Staphylococcus aureus* (*S. aureus*), and *Enterococcus* spp. are reported [5]. These microorganisms can survive on inanimate fomites under different conditions, even for extended periods, ranging from hours to months, thereby increasing the risk of infection transmission [6,7,8].

These pathogens are responsible for a wide range of healthcare-associated infections (HAIs), contributing to increased morbidity, mortality, and healthcare costs worldwide [9,10,11]. It is estimated that HAIs affect 6.5% of hospitalized patients in the European Union and 3.2% in the United States, with global prevalence likely increasing [12]. According to European epidemiological data, *P. aeruginosa*, *S. aureus*, and *Enterococcus* spp. are among the leading pathogens causing HAIs, especially due to the rising prevalence of antibiotic-resistant strains in recent years [13,14,15]. Such infections can lead to severe complications, prolonged hospitalization, intensive care treatment, and even death [16].

Given the significant burden these pathogens place on healthcare systems, timely detection and identification of microorganisms are essential to minimize the risk of transmission and enhance infection control efforts aimed at preventing the onset of disease and curbing the spread of antimicrobial resistance (AMR) [17,18,19].

Traditional culture-based microbiological methods, such as agar contact plates or swab inoculation, remain the gold standard for pathogen detection in hospital environmental surveillance [20,21,22]. However, they are time-consuming and require specialized equipment and skilled personnel, often providing results only after several days [23]. Moreover, additional steps, including biochemical and confirmatory tests, can extend the analysis time to over a week [24,25]. This delay is critical in high-risk healthcare settings, where rapid detection is essential to prevent outbreaks [26]. Additionally, the need to deliver samples to specialized laboratories further slows response times. Considering this, given the urgency for real-time monitoring, traditional methods are not ideal for timely interventions [27,28,29]. These limitations have prompted, in recent years, research for faster and more efficient alternative analytical methods [30,31].

In this context, loop-mediated isothermal amplification (LAMP) has emerged as a promising molecular technique for rapid and cost-effective pathogen detection and identification [32]. LAMP allows DNA amplification at a constant temperature (60–65 °C), eliminating the need for thermal cyclers and enabling the use of compact and affordable devices [33,34,35,36]. Its simplicity allows it to be performed with minimal training, without the need for highly qualified personnel, making it ideal for point-of-care, on-site applications or low-resource settings [37,38]. Results are typically available within 30 to 60 min, offering a much faster alternative to traditional culture-based methods or PCR [39,40]. This rapidity is particularly valuable in high-risk healthcare settings, where rapid pathogen identification is essential for infection control and timely intervention.

Since its development, LAMP has proven to be a rapid and user-friendly method for pathogen detection, achieving successful results, particularly in clinical diagnosis, agricultural applications, and food safety [41,42,43]. For this reason, with the growing emphasis placed on environmental monitoring as part of infection control programs, there is a clear opportunity to adapt and optimize LAMP for this critical purpose.

Considering this, this study aimed to optimize the use of LAMP as an innovative approach for the rapid detection of *P. aeruginosa*, *S. aureus*, and *Enterococcus* spp. on environmental surfaces. Although the systems used were originally developed for water quality monitoring, the objective was to adapt and validate them for surface contamination assessment. For this purpose, a comparison was conducted under controlled conditions with the gold-standard culture method, across varying pathogen concentrations and pre-incubation times to evaluate LAMP sensitivity, specificity, and applicability for environmental surveillance.

## 2. Results

### 2.1. Assessment of 10-Fold Serial Dilution Accuracy

To assess the accuracy of the 10-fold dilution process used to prepare bacterial suspensions from the initial 0.5 McFarland standards of the three pathogens involved in the study, viable colonies grown on TSA with 5% sheep blood agar plates were counted and identified after seeding 100 µL from each dilution.

Where colony growth exceeded the countable limit, the results were recorded as “greater than 100” (‘>100’) CFU/plate. The results from the three replicates of the conducted experiments are reported as the mean CFU ± standard deviation (sd) for each pathogen (Table S1). For all three pathogens tested, the highest concentrations, ranging from 1.5 × 10^8^ to 1.5 × 10^4^ CFU/mL, resulted in counts exceeding the measurable limit. As the concentration decreased further (from 1.5 × 10^3^ to 1.5 × 10^1^ CFU/mL), the number of colonies also decreased by a factor of ten. All negative control plates reported no colonies.

### 2.2. Comparison of the Two Different Sampling Methods

The cultural results obtained using TSA contact plates and swabs are summarized in Table S2 and reported as the mean of CFU/cm^2^ ± standard deviation (sd) for each pathogen. The mean and standard deviation values reported refer to the results obtained across the three biological replicates. The analysis of all surfaces inoculated with Ringer solution as a negative control showed no growth for both sampling methods. When colonies exceeded countable limits, the results were recorded as “greater than 4.17” (‘>4.17’) CFU/cm^2^.

For both sampling methods, *P. aeruginosa* and *Enterococcus faecalis* (*E. faecalis*) bacterial growth was detected down to a concentration of 1.5 × 10^2^, while for *S. aureus*, growth was detected down to a minimum concentration of 1.5 × 10^1^. The Wilcoxon test comparing the two sampling methods revealed a *p*-value of 0.72, 0.10, and 0.64 for *P. aeruginosa*, *S. aureus*, and *E. faecalis*, respectively.

### 2.3. Comparison Between Agar Contact Plates and Lamp Method

To evaluate the performance of the three different LAMP detection kits at the three different incubation times chosen (three, six, and nine hours), the results for each pathogen were compared with those obtained using the gold-standard culture-based method. The results of the contaminated surfaces analysis for *P. aeruginosa*, *S. aureus*, and *E. faecalis* using TSA contact plates and the LAMP detection kits are compared and summarized in Table 1. The applied pathogen dilution concentration (CFU/mL) refers to the concentration of bacterial suspension used to contaminate the test surfaces in duplicate. The culture-based method results are presented as the mean ± sd of CFU/contact plate detected during the three experimental replicates. When colony growth exceeded countable limits due to high density, the results were recorded as “greater than 100” (‘>100’) CFU/plate. By contrast, qualitative LAMP results, which were consistent across all three experimental replicates, are reported as positive (‘+’) or negative (‘−’) samples in relation to the three different incubation times studied (three, six, and nine hours).

The analysis of all the surfaces inoculated with Ringer solution as a negative control reported negative results for both the gold-standard cultural method and LAMP, for all the pathogens involved in the study.

For *P. aeruginosa*, the cultural method using TSA contact plates showed the presence of more than 100 CFU/plate at higher dilutions (from 1.5 × 10^8^ to 1.5 × 10^4^ CFU/mL), while lower dilutions resulted in fewer colonies, with none detected at 1.5 × 10^1^ CFU/mL or in the negative control. The LAMP assay showed positive results at higher dilutions (from 1.5 × 10^8^ to 1.5 × 10^4^ CFU/mL) regardless of the incubation time. At 1.5 × 10^3^ CFU/mL, the results were negative at three hours but turned positive at six and nine hours. Similarly, at 1.5 × 10^2^ CFU/mL, the results were negative at three and six hours but positive at nine hours. No positive results were observed at 1.5 × 10^1^ CFU/mL or in the negative control.

For *S. aureus*, the analysis by TSA contact plates consistently showed more than 100 CFU/plate at higher dilutions (from 1.5 × 10^8^ to 1.5 × 10^4^ CFU/mL). Lower dilutions resulted in fewer colonies, with approximately only 4 CFU at 1.5 × 10^1^ CFU/mL and none detected in the negative control. On the other hand, LAMP showed positive results at all dilutions and sample incubation times. No positive results were observed in the negative control.

Finally, for *E. faecalis*, the cultural TSA contact plate method showed more than 100 CFU/plate at higher dilutions (from 1.5 × 10^8^ to 1.5 × 10^5^ CFU/mL), while at lower concentrations, the number of colonies decreased proportionally, with none detected at 1.5 × 10^1^ CFU/mL and in the negative control. On the other hand, LAMP showed positive results after three hours of incubation up to a pathogen concentration of 1.5 × 10^5^ CFU/mL, while at both six and nine hours of incubation, LAMP was able to detect the presence of the pathogen up to a concentration of 1.5 × 10^2^ CFU/mL. No positive results were observed at 1.5 × 10^1^ CFU/mL or in the negative control.

The experimental conditions tested permit the calculation of a limit of detection (LOD) for each microorganism at all incubation times. For *P. aeruginosa*, the LOD was 1.5 × 10^4^ CFU/mL, 1.5 × 10^3^ CFU/mL, and 1.5 × 10^2^ CFU/mL at 3, 6, and 9 h, respectively. For *S. aureus*, the LOD was 1.5 × 10^1^ CFU/mL across all incubation times. For *E. faecalis*, the LOD improved from 1.5 × 10^5^ CFU/mL at 3 h to 1.5 × 10^2^ CFU/mL at 6 and 9 h.

Additionally, based on the results obtained, the Se, Sp, Acc, PPV, NPV, Pre, Rec, F1, and Bal Acc were calculated for each LAMP detection kit and the incubation times studied (three, six, and nine hours) (Table S3). The culture-based method was used as the reference standard. To compare LAMP with the TSA contact plate method, the cultural results were considered as positive or negative samples, based on the presence or absence of bacterial colonies.

The results showed that the Se of LAMP varied with different pathogens and incubation times. For *P. aeruginosa*, the Se increased from 0.71 at three hours to 0.86 at six hours, reaching 1.00 at nine hours. In contrast, the LAMP detection kit for *S. aureus* consistently showed a Se of 1.00 regardless of sample incubation. For *Enterococcus* spp., the Se was initially lower (0.57) at three hours but improved to 1.00 at six and nine hours. Conversely, the Sp remained consistently high at 1.00 for all pathogens and incubation times.

The Acc followed a similar trend to the Se. For *P. aeruginosa*, the Acc improved from 0.78 at three hours to 0.89 at six hours, reaching 1.00 at nine hours. For *S. aureus*, the Acc was 1.00 at all incubation times. For *Enterococcus* spp., the Acc increased from 0.67 at three hours to 1.00 at six and nine hours.

The PPV was 1.00 for all pathogens and incubation times. On the other hand, the NPV for *P. aeruginosa* increased from 0.50 at three hours to 0.67 at six hours and reached 1.00 at nine hours, while for *S. aureus*, it showed a value of 1.00 at all incubation times. For *Enterococcus* spp., the NPV started from 0.40 at three hours and improved to 1.00 at six and nine hours.

The Pre was consistently high at 1.00 for all pathogens and incubation times. In contrast, both the F1-score and Bal Acc followed the trend of the Se. For *P. aeruginosa*, both metrics improved from 0.83 and 0.86 at three hours to 0.92 and 0.93 at six hours, respectively, reaching 1.00 at nine hours. For *S. aureus* the metrics were consistently 1.00 at all incubation times, while for *Enterococcus* spp., they improved from 0.57 and 0.67 at three hours to 1.00 at six and nine hours.

Overall, the results showed that at nine hours of incubation, all metrics (Se, Sp, Acc, PPV, NPV, Pre, F1, and Bal Acc) reached a value of 1.00 for all pathogens.

### 2.4. Concordance of LAMP Results at the Three Different Incubation Times

The level of results concordance of each LAMP detection kit at the three different incubation times studied (three, six, and nine hours) was assessed using Cohen’s kappa coefficient (k), which accounts for the agreement occurring by chance. The results are summarized in Table 2.

For *P. aeruginosa*, the k value comparing three to six hours and six to nine hours was quite similar (0.77 and 0.73, respectively). In contrast, the k value comparing three to nine hours was lower (0.53). For *S. aureus*, the k values were consistently 1.00 for all comparisons. For *Enterococcus* spp., the k values comparing three to six hours and three to nine hours were both 0.37. In contrast, the k value comparing six to nine hours was 1.00.

## 3. Discussion

The research of rapid detection methods for nosocomial pathogens is an essential issue in the context of a disease-preventive approach. The presence of these microorganisms in high-risk environments, such as hospitals and healthcare facilities, poses a significant risk to public health [44,45]. For this reason, routine microbiological screening of these critical environments is fundamental for preventing infection cases and minimizing disease rates [46]. However, the techniques commonly used for this purpose are often too labor-intensive and time-consuming. Therefore, the search for alternative and faster methods is becoming increasingly important [47].

In recent years, LAMP has emerged as a rapid and innovative technique for pathogen detection across various fields, with research on this topic exponentially increasing [48]. Several studies have already documented the effectiveness of LAMP in clinical diagnostics, for instance, in the detection of *Mycobacterium tuberculosis*, *Neisseria meningitidis*, and HIV, as well as in food safety, where it has been used to identify foodborne pathogens such as *Salmonella* spp., *Listeria monocytogenes*, and *Anisakis* spp. [49,50,51,52,53,54]. However, its application in environmental monitoring remains relatively underexplored, with only a few studies investigating its use in surface water surveillance, water quality assessment, or the environmental detection of SARS-CoV-2 [55,56,57,58,59].

Therefore, although LAMP was developed over two decades ago and has proven to be a robust and accessible technique, its limited application in environmental monitoring may be attributed to the historical dominance of culture-based methods in this field, as well as to the lack of standardized protocols and regulatory frameworks for molecular tools in routine surface surveillance.

For this reason, and especially considering the growing relevance of HAIs, optimizing rapid methods for environmental surveillance of nosocomial pathogens is a key public health objective. In this context, this study aimed to evaluate the performance of three different LAMP detection kits as a rapid and reliable alternative for detecting *P. aeruginosa*, *S. aureus*, and *E. faecalis* on contaminated surfaces. These microorganisms were selected due to their high prevalence in nosocomial infections and the critical need to ensure their complete absence in high-risk healthcare settings [60,61]. In line with the limited availability of LAMP applications for environmental surface monitoring discussed above, the lack of commercially available kits specifically designed for this purpose led us to adapt and optimize kits originally developed for water testing.

To validate the use of these kits for surface applications, the LAMP method was compared with the reference method under controlled laboratory conditions. Firstly, the accuracy of the 10-fold serial dilution process used to prepare the bacterial suspensions was assessed. The enumeration of viable colonies confirmed that the dilution factors were correctly applied for all three pathogens. The consistency of the results across the three experimental replicates, as indicated by low standard deviations, further confirmed the reliability of the dilution process. This step was essential to ensure the validity of subsequent analyses, as any error in the initial bacterial concentrations could have compromised the comparison between methods.

Moreover, negative control plates showed no colony growth, confirming the absence of external contamination.

In the second phase, using the reference dilution for each pathogen, two sterile 24 cm^2^ surfaces were contaminated in duplicate. One surface was sampled using the agar contact plate method (the gold standard), while the other was sampled, for LAMP analysis, using a swab.

Furthermore, since previous studies have shown that different sampling methods can yield varying microbial recovery rates [62,63], a comparison was conducted between the two approaches—TSA contact plates and swabs—to minimize potential bias in the study. The absence of statistically significant differences between the two methods for *P. aeruginosa* (*p* = 0.72), *S. aureus* (*p* = 0.10), and *E. faecalis* (*p* = 0.64) suggests that both are suitable for this type of analysis. Additionally, the lack of growth in the negative controls further confirmed the reliability of the experimental setup.

Following sampling, the contact plates were directly incubated at 36 °C for 48 h, while the swabs were incubated in pre-enrichment broth. Despite the manufacturer’s recommendation of an 18 h sample incubation before LAMP analysis, shorter incubation times—3, 6, and 9 h—were evaluated to improve the responsiveness of surface hygiene assessments. This approach aimed to support the use of LAMP as a real-time monitoring tool. The results obtained were then compared to those of the gold-standard culture method.

For *P. aeruginosa*, the best performance was observed after nine hours of sample incubation. The Se increased from 0.71 at 3 h to 1.00 at 9 h, indicating that longer incubation times enhanced the ability of the test to detect true positives. In contrast, the Sp, PPV, and Pre remained consistently high at 1.00 across all time points, confirming the absence of false positives. All the other performance metrics, including the Acc, NPV, F1-score, and Bal Acc, also improved progressively, reaching 1.00 at nine hours. These results suggested that, despite the LAMP results being highly specific for *P. aeruginosa*, a longer incubation period is necessary to achieve optimal sensitivity.

For *S. aureus*, all performance metrics (Se, Sp, Acc, PPV, NPV, Pre, F1-score, and Bal Acc) were consistently 1.00 at all incubation times. This indicates that the LAMP assay was highly effective in detecting *S. aureus* even after only three hours of incubation, demonstrating both rapidity and reliability.

For *E. faecalis*, performance improved significantly between three and six hours of incubation. At three hours, the sensitivity was lower, but from six hours onwards, all metrics reached 1.00. This suggested that a minimum of six hours of incubation is required to ensure accurate detection of *E. faecalis*, with both true positives and true negatives correctly identified.

All surfaces inoculated with Ringer solution as the negative controls yielded negative results with both the culture-based method and LAMP, further confirming the specificity and reliability of the experimental setup.

Finally, the concordance of the LAMP results at different incubation times was evaluated using Cohen’s kappa coefficient. Substantial agreement was found for *P. aeruginosa* between three and six hours (k = 0.77) and six and nine hours (k = 0.73), indicating that LAMP provided consistent results within these time frames. However, the moderate agreement between three and nine hours (k = 0.53) suggested that a shorter incubation time might introduce some inconsistencies. For *S. aureus*, the perfect agreement (k = 1.00) across all comparisons indicated a high LAMP reliability and consistency for detecting the pathogen at any of the studied incubation times. For *E. faecalis*, the fair agreement between three and six hours (k = 0.37) and between three and nine hours (k = 0.37) indicated that the LAMP detection kits were less consistent after only three hours of sample incubation. However, the perfect agreement between six and nine hours (k = 1.00) suggested that a period of incubation from six hours onwards was sufficient to obtain a reliable detection of *E. faecalis*.

In conclusion, the overall performance metrics suggested that the 18 h incubation time recommended by the manufacturer may not be necessary. Shorter incubation periods—specifically six to nine hours—were sufficient to achieve reliable results, significantly reducing the total time required for pathogen detection. Although *S. aureus* showed excellent performance even at three hours, for practical implementation in real-world workflows, an incubation time between six and nine hours is recommended. 

This strategy can indeed offer practical advantages in terms of workflow optimization. By standardizing the incubation time to a single interval (six to nine hours), it becomes possible to process each surface sample with a single swab and perform DNA extraction only once. The extracted DNA can then be aliquoted and used simultaneously across multiple LAMP kits targeting different pathogens. This not only reduces the handling time and reagent consumption but also simplifies the overall procedure, making it more suitable for routine implementation in real-world settings.

The high efficacy of LAMP recorded for the detection of all three pathogens involved in this study perfectly aligned with the latest findings and the scientific literature [64]. Although LAMP-based applications are extensively documented and widely used in various diagnostic fields, their application for environmental surveillance, particularly on surfaces and in air, which are critical for nosocomial infection control, remains largely unexplored. As a result, the data obtained in this study were compared with findings from related fields such as human infection control, food safety, and wastewater monitoring. For instance, Brandolini et al. recently demonstrated the high diagnostic performance of LAMP in detecting sexually transmissible pathogens, while Mellikeche et al. assessed its effectiveness in detecting *Aspergillus* contamination in food samples, emphasizing its rapidity and simplicity [65,66].

Additionally, the use of this technology directly on site, as suggested by several other studies [67,68,69], can further reduce analysis times, making it an even more effective tool for real-time pathogen surveillance. This capability could enable healthcare facilities to conduct more frequent and timely environmental screenings, thereby improving overall infection control measures.

Although the results obtained are promising, it is important to highlight that, like other molecular techniques, the limit of the LAMP technique is the detection of microbial DNA, regardless of the viability of the microorganisms. This aspect is particularly relevant in hospital environments, where disinfectants are widely used and may inactivate pathogens without degrading their DNA. As a result, LAMP could potentially detect DNA from non-viable organisms, leading to an overestimation of the actual microbiological risk. This limitation should be carefully considered when interpreting results in real-world applications, especially in the context of infection prevention and control strategies.

Nevertheless, due to its high sensitivity, specificity, and rapid turnaround time, LAMP could represent a valuable first-line screening tool in routine environmental surveillance, allowing for timely decision-making and prioritization of confirmatory culture-based analyses. Moreover, the cost savings in terms of reduced time, materials, equipment, and trained personnel for colony subculture and identification should not be underestimated.

Additionally, considering that the study’s controlled conditions may not fully reflect real-world hospital environments, further research is needed to validate these findings. Future studies should focus on testing LAMP in real healthcare facility scenarios to determine its practical utility and identify any potential challenges.

## 4. Materials and Methods

### 4.1. Bacteria Dilution and Surface Contamination

To compare LAMP with the gold-standard culture-based method, three different 0.5 McFarland bacterial suspensions (1.5 × 10^8^ CFU/mL) of, respectively, *P. aeruginosa* (ATCC 10145), *S. aureus* (ATCC 23235), and *E. faecalis* (ATCC 29212) reference strains were prepared in a total volume of 10 mL of sterile Ringer solution (Oxoid, Basingstoke, UK). Each bacterial suspension was then 10-fold serially diluted up to a final concentration of approximately 1.5 × 10^1^ CFU/mL.

To assess the accuracy of the diluting process, 100 µL of each diluted solution was plated onto tryptone soy agar (TSA) with 5% sheep blood agar (Thermo Fisher Diagnostics, Basingstoke, UK). Additionally, as a negative control, 100 µL of Ringer solution was also seeded on TSA with 5% sheep blood agar plate to assess its sterility. All plates were then incubated at 35 ± 2 °C for 48 h. The viable grown colonies were then enumerated to check whether the dilution factor was respected. Results were recorded as the number of colony-forming units (CFUs) per plate (CFU/plate).

After dilution, sterile surfaces of 24 cm^2^, marked on polystyrene Petri dishes (Artiglass, Padova, Italy), were contaminated in duplicate with 250 µL of each bacteria-diluted suspension. The total volume was evenly spread as 2 µL tiny droplets across each sterile test surface by using a pipette. Contamination was performed under strictly controlled conditions: each surface was contaminated and left to dry for about one hour under a laminar flow hood before sampling. The size of the surface to be contaminated was chosen to exactly match the area of a contact plate with a diameter of 55 mm. Additionally, sterile 24 cm^2^ test surfaces were inoculated in duplicate with 250 µL of Ringer solution (Oxoid, Basingstoke, UK) and used as a negative control. For each pair of contaminated surfaces, one was sampled and analyzed using the standard contact plate method (UNI EN 17141:2021), while the other was sampled with SRK^®^ FLOQSwabs^®^ (Copan Italia, Brescia, Italy) and then analyzed using LAMP (Enbiotech, Palermo, Italy).

### 4.2. Agar Contact Plate Method

One of each pair of contaminated surfaces was sampled and analyzed following UNI EN 17141:2021, which describes the best practices for controlling microbiological contamination in all clean controlled environments [70]. The agar contact plate method was chosen as the gold standard for the analysis of flat surfaces.

Tryptone soy agar (TSA) contact plates (Pharmamedia, Leimen, Germany) with a 55 mm diameter and a 24 cm^2^ area were applied on each round test contaminated surface and held in place for ten seconds with uniform and steady pressure. The same procedure was also applied to one of the two negative control surfaces to detect any potential unwanted contamination. Subsequently, all plates were incubated at 35 ± 2 °C for 48 h. Following the incubation period, colonies grown on each plate were enumerated and then identified using the Matrix-Assisted Laser Desorption/Ionization—Time of Flight (MALDI) Biotyper system^®^ (Bruker Daltonics, Bremen, Germany). Results were recorded as the number of colony-forming units (CFUs) per contact plate (CFU/contact plate).

### 4.3. Loop-Mediated Isothermal Amplification (LAMP) Analysis

The other contaminated surfaces were analyzed using LAMP. Three different LAMP detection kits were employed for the analysis: one for *P. aeruginosa*, one for *S. aureus*, and one for *Enterococcus* spp., all provided and commercialized by Enbiotech, Palermo, Italy.

Surface sampling was conducted using FLOQSwabs^®^. Each sample was collected by rubbing and rotating a regular, moistened swab in three different directions over the 24 cm^2^ test surface. The swabs were then placed inside sterile tubes containing 2.5 mL of SRK^®^ transport medium (Copan Italia, Brescia, Italy). The same procedure was also applied to the second negative control surface.

To evaluate the effectiveness of the swab sampling, the tubes were vortexed, and 100 µL of the SRK^®^ transport medium from each sample was plated on TSA with 5% sheep blood agar plates (Thermo Fisher Diagnostics, Basingstoke, UK), which were incubated at 35 ± 2 °C for 48 h. The viable colonies grown on plates were enumerated and then identified using MALDI Biotyper^®^ (Bruker Daltonics, Bremen, Germany). The results were recorded as CFU/100 µL.

Following seeding, as stated within the LAMP protocol, 2 mL of the nutrient broth present in the kit was added to every swab sample. The swabs were then incubated at 35 ± 2 °C. Although the manufacturer recommends an 18 h incubation, to reduce analysis time and assess the effectiveness of LAMP even at shorter incubation periods, 1.4 mL aliquots from each sample were collected in sterile 1.5 mL tubes after 3, 6, and 9 h of incubation.

DNA extraction was performed according to the manufacturer’s protocol (Enbiotech, Palermo, Italy). All collected aliquots were centrifuged at 13,000× *g* for five minutes, and the supernatant was discarded. The pellet was resuspended in 200 µL of the extraction buffer (provided by the LAMP kits) and homogenized by vortexing. The samples were then incubated at 95 ± 2 °C for ten minutes to facilitate bacterial DNA extraction. Lastly, the samples were centrifuged for one minute at 13,000× *g* to separate cell debris from the supernatant, which was used as the template.

The template DNA was then added, for each sample, to the LAMP Mix (consisting of enzyme, Magnesium chloride, nucleotides, and reaction buffers) inside the Primer Mix tubes (different for each kit), containing dried microorganism-specific primers at the bottom. LAMP mix and Primer Mix tubes were both provided by the LAMP kits involved in the study. Specifically, according to manufacturer protocols, (i) 6 µL of DNA was added to 14 µL of LAMP mix for the *P. aeruginosa* detection kit, (ii) 6 µL of DNA was added to 14 µL of LAMP mix for the *S. aureus* detection kit, and (iii) 3 µL of DNA was added to 22 µL of LAMP mix for the *Enterococcus* spp. detection kit. All the components, as well as the sequence of primers contained in the kit, are covered by the manufacturer’s patent.

Finally, all tubes were placed into the portable ICGENE Plus device (Enbiotech, Palermo, Italy) to start the amplification process for 60 min at 65 °C. Positive and negative DNA controls, included in each LAMP kit, were also contextually analyzed. The system consists of a tablet with a dedicated application (ICGENE app version 3.9.10) that allows, thanks to a fluorometer, the real-time display of the sigmoid amplification curve and the automatic interpretation of qualitative results, which were expressed as positive (‘+’) or negative (‘−’) samples.

All experiments were conducted in three independent biological replicates for each microorganism. Each replicate included the complete procedure of surface contamination, sampling, and analysis using both the gold-standard method and LAMP.

### 4.4. Comparison Between the Two Different Sampling Methods

To ensure that the LAMP results were not biased due to differences in the sampling method used, for each of the involved pathogens, a comparison was made between the results obtained using the agar contact plate method and the seeding of SRK^®^ transport medium, after surface sampling and before LAMP analysis. Agar contact plates are considered the gold standard for flat surface sampling, while swabs were required for the LAMP protocol due to the need for liquid-phase processing. To make the two sampling techniques comparable, the measurement units of the results (CFU/contact plate and CFU/100 µL, respectively) were converted to a single unit of measurement (CFU/cm^2^).

To achieve this, the results from the agar contact plates were divided by the total sampled surface area (24 cm^2^), while those from the seeding of the swab transport medium were first adjusted to the total volume of the transport liquid (2.5 mL) and then divided by the total sampled surface area (24 cm^2^).

### 4.5. Statistical Analysis

The statistical analyses were performed using R software version 4.4.1 [71]. The comparison of the two sampling methods was conducted using the Shapiro–Wilk test and the Wilcoxon test.

The results obtained using the culture-based method and LAMP technique were analyzed as positive or negative units. In detail, the sensitivity (Se), specificity (Sp), accuracy (Acc), positive predictive value (PPV), negative predictive value (NPV), precision (Pre), F1-score (F1), and balanced accuracy (Bal Acc) were calculated for each LAMP kit involved in the study, considering the gold-standard culture-based method as the reference [72]. The formulas used for statistical analysis are reported in Supplementary Materials (SM).

Moreover, for each LAMP kit, the level of concordance between the results obtained at the three different incubation times studied (three, six, and nine hours) was calculated, using the standardized Cohen’s coefficient (k) [73].

## 5. Conclusions

Within the expanding context of research on LAMP applications, this study highlighted the significant potential of this technique as a rapid and reliable alternative to traditional culture-based methods for detecting nosocomial pathogens such as *P. aeruginosa*, *S. aureus*, and *E. faecalis* on environmental surfaces. The high sensitivity and specificity of LAMP, combined with its ability to deliver results within 6 to 9 h, offer substantial advantages for infection control in healthcare settings.

The rapid detection enabled by LAMP allows for timely interventions, optimizing the use of resources and enhancing patient safety. These operational efficiencies translate into a lower overall cost per analysis, making LAMP a practical and scalable solution for routine environmental surveillance in healthcare environments. Moreover, by integrating LAMP into routine environmental monitoring, healthcare facilities could improve pathogen surveillance, disinfection strategies, and better protect both patients’ and healthcare workers’ health.

## Figures and Tables

**Table 1:** Comparison of TSA contact plates and LAMP results for the detection of *P. aeruginosa* (ATCC 10145), *S. aureus* (ATCC 23235), and *E. faecalis* (ATCC 29212).

	*P. aeruginosa*				*S. aureus*				*E. faecalis*			
Applied Pathogen Dilution (CFU/mL)	TSA Contact Plates (CFU/Plate)	LAMP (+/−)			TSA Contact Plates (CFU/Plate)	LAMP (+/−)			TSA Contact Plates (CFU/Plate)	LAMP (+/−)		
		3 h	6 h	9 h		3 h	6 h	9 h		3 h	6 h	9 h
1.5 × 10^8^	>100	+	+	+	>100	+	+	+	>100	+	+	+
1.5 × 10^7^	>100	+	+	+	>100	+	+	+	>100	+	+	+
1.5 × 10^6^	>100	+	+	+	>100	+	+	+	>100	+	+	+
1.5 × 10^5^	>100	+	+	+	>100	+	+	+	>100	+	+	+
1.5 × 10^4^	107.67 ± 14.57	+	+	+	>100	+	+	+	96.33 ± 5.86	−	+	+
1.5 × 10^3^	9.67 ± 2.08	−	+	+	96.00 ± 9.64	+	+	+	16.00 ± 3.00	−	+	+
1.5 × 10^2^	1.00 ± 0.00	−	−	+	17.00 ± 2.00	+	+	+	3.00 ± 1.00	−	+	+
1.5 × 10^1^	0.00 ± 0.00	−	−	−	4.00 ± 1.00	+	+	+	0.00 ± 0.00	−	−	−
Negative control	0.00 ± 0.00	−	−	−	0.00 ± 0.00	−	−	−	0.00 ± 0.00	−	−	−

Note: the value > 100 indicates counts exceeding 100 CFU per plate. “+” for positive, “−” for negative LAMP results.

**Table 2:** Cohen’s kappa coefficient (k) for LAMP result concordance at three, six, and nine hours for *P. aeruginosa*, *S. aureus*, and *Enterococcus* spp.

		LAMP Detection Kit		
		*P. aeruginosa*	*S. aureus*	*Enterococcus* spp.
**k**	3 h vs. 6 h	0.77	1.00	0.37
	3 h vs. 9 h	0.53	1.00	0.37
	6 h vs. 9 h	0.73	1.00	1.00

## Data Availability

All relevant data are provided in the manuscript. The corresponding author can provide additional data as a reasonable request.

## Supplementary Materials

The following supporting information can be downloaded at: https://www.mdpi.com/article/10.3390/ijms26135933/s1.
