# Supplementary material for: Optimization of Loop-Mediated Isothermal Amplification (LAMP) for the Rapid Detection of Nosocomial Pathogens on Environmental Surfaces
Source: Int J Mol Sci. 2025 Jun 20;26(13):5933. doi: 10.3390/ijms26135933 (PMC12249420; doi:10.3390/ijms26135933)
Supplement: Supplementary file 1 [file ijms-26-05933-s001.zip › ijms-3647572-supplementary.pdf]

(LAMP) for the rapid detection of nosocomial pathogens on environmental surfaces.

Marino F [1], Bonincontro C [1], Caligaris L [1], Bellucci L [2], Derelitto C [1], Girolamini L [1] and Cristino S [1,*]

[1] Department of Biological, Geological, and Environmental Sciences, University of Bologna, 40126, Bologna, Italy
[2] Class S.r.l., 40054, Budrio, Italy
*Correspondence: Address correspondence to Sandra Cristino, sandra.cristino@unibo.it

The formulas used for statistical analysis are provided below:

- Sensitivity (Se) = TP/TP+FN
- Specificity (Sp) = TN/TN+FP
- Accuracy (Acc) = TP+TN/TP+TN+FP+FN
- Positive Predictive Value (PPV) = TP/TP+FP
- Negative Predictive Value (NPV) = TN/TN+FN
- F1-score (F1) = 2·(Precision·Recall)/(Precision+Recall)
- Balanced Accuracy (Bal Acc) = (Sensitivity+Specificity)/2

Table S1. Number of CFU/plate for *P. aeruginosa* (ATCC 10145), *S. aureus* (ATCC 23235), and *E. faecalis* (ATCC 29212) 10-fold diluted bacterial suspensions.

		Pathogen concentration (CFU/mL)							
		$1.5x10^8$	$1.5x10^7$	$1.5x10^6$	$1.5x10^5$	$1.5x10^4$	$1.5x10^3$	$1.5x10^2$	$1.5x10^1$	Negative control
CFU/plate (mean±sd)	*P. aeruginosa*	>100	>100	>100	>100	>100	136.33±9.55	14.33±2.52	0.67±0.58	0.00±0.00
	S. aureus	>100	>100	>100	>100	>100	109.67±5.96	11.00±2.00	1.67±0.58	0.00±0.00
	E. faecalis	>100	>100	>100	>100	>100	103.67±7.37	9.00±1.00	0.67±0.58	0.00±0.00

Table S2. Comparison of cultural results obtained by using TSA contact plates and swabs as different sampling methods for *P. aeruginosa* (ATCC 10145), *S. aureus* (ATCC 23235), and *E. faecalis* (ATCC 29212).

	Applied pathogen concentration (CFU/mL)										
	Sampling method	$1.5x10^8$	$1.5x10^7$	$1.5x10^6$	$1.5x10^5$	$1.5x10^4$	$1.5x10^3$	$1.5x10^2$	$1.5x10^1$	Negative control	*p*-value
P. aeruginosa (CFU/cm^2)	Contact plate	>4.17	>4.17	>4.17	>4.17	4.49±0.61	0.40±0.09	0.04±0.00	0.00±0.00	0.00±0.00	0.72
	Swab	>4.17	>4.17	>4.17	>4.17	3.89±0.24	0.42±0.00	0.14±0.24	0.00±0.00	0.00±0.00	
S. aureus (CFU/cm^2)	Contact plate	>4.17	>4.17	>4.17	>4.17	>4.17	4.00±0.40	0.71±0.08	0.17±0.04	0.00±0.00	0.10
	Swab	>4.17	>4.17	>4.17	>4.17	>4.17	4.58±0.83	0.83±0.42	0.28±0.24	0.00±0.00	
E. faecalis (CFU/cm^2)	Contact plate	>4.17	>4.17	>4.17	>4.17	4.01±0.24	0.67±0.13	0.13±0.04	0.00±0.00	0.00±0.00	0.64
	Swab	>4.17	>4.17	>4.17	>4.17	4.17±1.10	0.83±0.42	0.28±0.24	0.00±0.00	0.00±0.00	

Note: the value >4.17 CFU/cm^2 indicates counts exceeding 100 CFU, normalized to a 24 cm^2 test surface

Table S3. Performance metrics of LAMP detection kits at the different incubation times. Sensitivity (Se), specificity (Sp), accuracy (Acc), positive predictive value (PPV), negative predictive value (NPV), precision (Pre), F1-score (F1), and balanced accuracy (Bal Acc).

LAMP detection kit		Se	Sp	Acc	PPV	NPV	Pre	F1	Bal Acc
P. aeruginosa	3h	0.71	1.00	0.78	1.00	0.50	1.00	0.83	0.86
	6h	0.86	1.00	0.89	1.00	0.67	1.00	0.92	0.93
	9h	1.00	1.00	1.00	1.00	1.00	1.00	1.00	1.00
S. aureus	3h	1.00	1.00	1.00	1.00	1.00	1.00	1.00	1.00
	6h	1.00	1.00	1.00	1.00	1.00	1.00	1.00	1.00
	9h	1.00	1.00	1.00	1.00	1.00	1.00	1.00	1.00
Enterococcus spp.	3h	0.57	1.00	0.67	1.00	0.40	1.00	0.73	0.79
	6h	1.00	1.00	1.00	1.00	1.00	1.00	1.00	1.00
	9h	1.00	1.00	1.00	1.00	1.00	1.00	1.00	1.00
